# Comprehensive Strain-Level Analysis of the Gut Microbe Faecalibacterium prausnitzii in Patients with Liver Cirrhosis

**DOI:** 10.1128/mSystems.00775-21

**Published:** 2021-08-03

**Authors:** Yaowen Chen, Pu Liu, Runyan Liu, Shuofeng Hu, Zhen He, Guohua Dong, Chao Feng, Sijing An, Xiaomin Ying

**Affiliations:** a Center for Computational Biology, Beijing Institute of Basic Medical Sciences, Beijing, China; Qingdao Institute of Bioenergy and Bioprocess Technology, Chinese Academy of Sciences

**Keywords:** liver cirrhosis, *Faecalibacterium prausnitzii*, within-species variation, species heterogeneity, strain diversity, strain-level analysis, single-nucleotide polymorphisms, gut microbiome, human metagenomics

## Abstract

Liver cirrhosis (LC) has been associated with gut microbes. However, the strain diversity of species and its association with LC have received little attention. Here, we constructed a computational framework to study the strain heterogeneity in the gut microbiome of patients with LC. Only Faecalibacterium prausnitzii shows different single-nucleotide polymorphism (SNP) patterns between the LC and healthy control (HC) groups. Strain diversity analysis discovered that although most F. prausnitzii genomes are more deficient in the LC group than in the HC group at the strain level, a subgroup of 19 *F. prausnitzii* strains showed no sensitivity to LC, which is inconsistent with the species-level result. The functional differences between this subgroup and other strains may involve short-chain fatty acid production and chlorine-related pathways. These findings demonstrate functional differences among *F. prausnitzii* subgroups, which extend current knowledge about strain heterogeneity and relationships between *F. prausnitzii* and LC at the strain level.

**IMPORTANCE** Most metagenomic studies focus on microbes at the species level, thus ignoring the different effects of different strains of the same species on the host. In this study, we explored the different microbes at the strain level in the intestines of patients with liver cirrhosis and of healthy people. Previous studies have shown that the species Faecalibacterium prausnitzii has a lower abundance in patients with liver cirrhosis than in healthy people. However, our results found multiple *F. prausnitzii* strains that do not decrease in abundance in patients with liver cirrhosis. It is more sensitive to select the appropriate strains as indicators to distinguish between the disease and the control samples than to use the entire species as an indicator. We clustered multiple *F. prausnitzii* strains and discuss the functional differences of different clusters. Our findings suggest that more attention should be paid to metagenomic studies at the strain level.

## INTRODUCTION

The gut microbiome has been associated with numerous diseases, including inflammatory bowel disease (IBD) (1), asthma (2), obesity (3), diabetes mellitus (4, 5), cardiovascular disease (6), Parkinson’s disease (7), and colorectal cancer (8). The development of DNA sequencing and bioinformatics tools has facilitated systematic investigation of the human gut microbiota and such disease associations. Many metagenomic studies have been performed to explore microbial communities at shallow levels, such as at the genus level to obtain 16S rRNA sequencing data and at the species level to obtain shotgun sequencing data (1–6, 8, 9). However, strain-level analysis is essential for the study of associations between microbes and diseases, as strains are the basic functional units that communicate with hosts.

To examine strain diversity, genomic variations, which include single-nucleotide polymorphisms (SNPs), short insertions/deletions, and structural variation among metagenomes, can be investigated first to help researchers focus on heterogeneous species. Schloissnig et al. (10) described the genomic variation landscape of the healthy human gut microbiome and found that subjects exhibited individual and temporal stability of SNP variation patterns, despite considerable changes in gut microbiota composition. In addition, strain-level variation in the microbiomes of diabetic wounds has been found to be associated with clinical outcomes (11).

Liver cirrhosis (LC) is the end stage of liver disease, occurring after decades of inflammation and fibrosis, and is among the most common causes of morbidity and mortality worldwide (12). Nonalcoholic fatty liver disease/nonalcoholic steatohepatitis has become the most common etiology of chronic liver disease, especially in those with diabetes (13). Gut-derived bacteria, along with their metabolites, nutrients, and other signals, are delivered to the liver via portal circulation. The liver plays a crucial role in defense against gut-derived materials (14). Enteric dysbiosis is involved in the progression of LC, and alteration of the gut microbiota has been shown to be an important factor in complications of end-stage liver cirrhosis, such as spontaneous bacterial peritonitis (15) and hepatic encephalopathy (16).

Among human gut microbes, Faecalibacterium prausnitzii was reported to be insufficiently abundant in the guts of patients with LC compared with those of healthy controls (HCs). *F*. *prausnitzii*, which is among the most common species in the adult human gastrointestinal tract, is also related to conditions such as Crohn’s disease (CD) (17), type 2 diabetes (18), and irritable bowel syndrome (IBD) (19). Recently, *F*. *prausnitzii* has also been related to coronavirus disease 2019 (COVID-19) (20). However, most studies of *F. prausnitzii* have been performed at the species level. Although the genomic heterogeneity of *F. prausnitzii* has been noted previously (18, 19, 21–25), large-scale, comprehensive research of *F. prausnitzii* strain diversity in the context of diseases, especially that of LC, is still lacking. In this study, we conducted a strain-level analysis of the gut metagenomes in LC and HC groups.

## RESULTS

### A framework of SNP analysis of disease-related microbes and strain diversity estimate.

**FIG 1:**
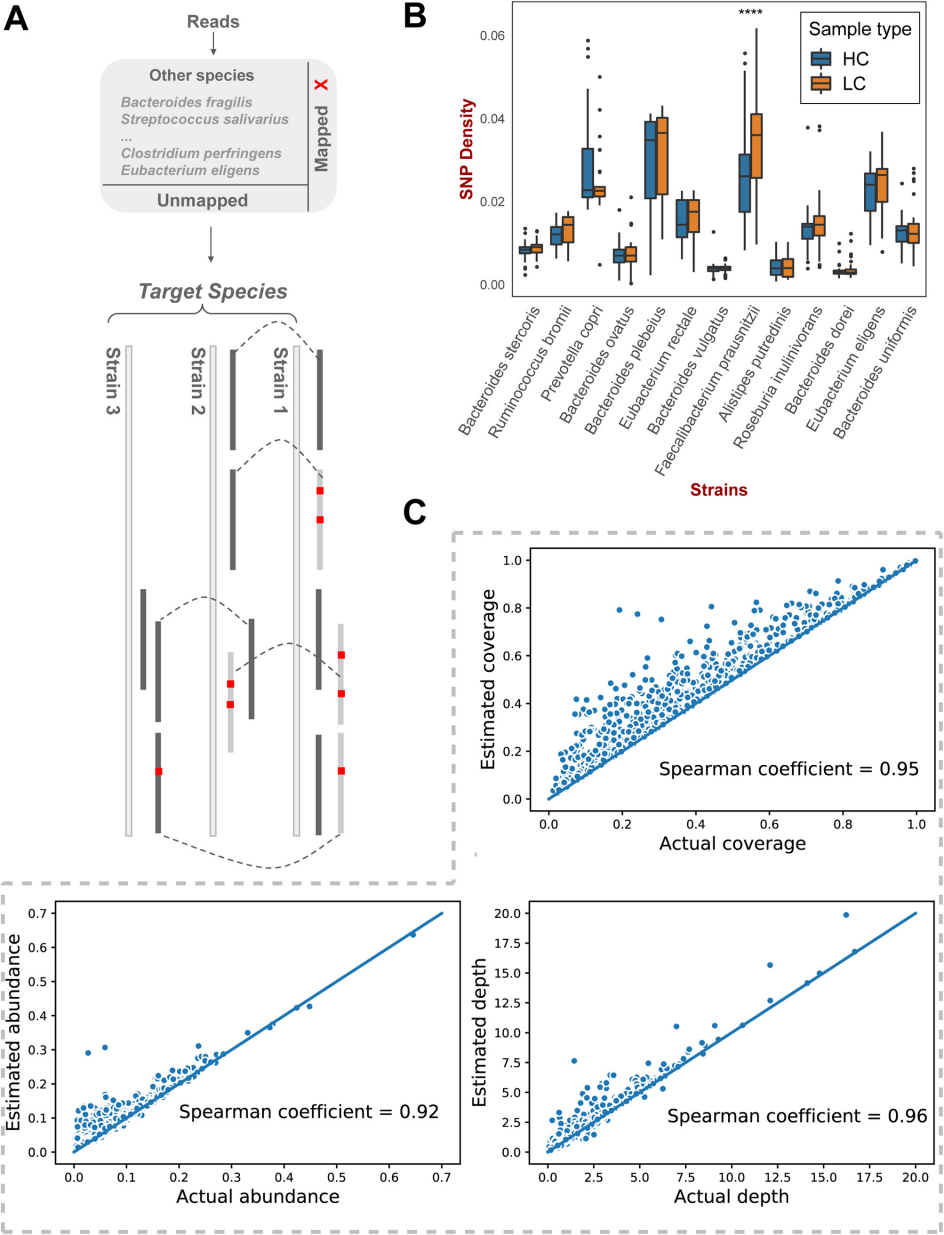
Framework of strain diversity analysis of disease-related microbes. (A) Interpretive pipeline of our strain diversity analysis tool. Red dots represent mismatches against the reference, short straight lines represent reads, black reads were assigned to the genomes below them, gray reads were assigned to other genomes, and dashed lines connect the same reads. (B) Differences in single-nucleotide polymorphism (SNP) density between the healthy control and liver cirrhosis groups for 13 prevalent strains. (C) Performance of our strain diversity analysis tool with synthetic data. (Top) Correlations between actual coverages and estimated coverages. (Bottom left) Correlations between actual abundances and estimated abundances. (Bottom right) Correlations between actual depths and estimated depths.

In order to discover the strain diversity of microbes in human gut and the possible association between strain heterogeneity and diseases, we constructed a two-step analysis framework. First, we tried to find microbes that may have differences in strain diversity between the disease group and the control group. This step was completed using the metagenomic SNP analysis pipeline (see Fig. S1 in the supplemental material). Second, for the microbes with different SNP patterns between the disease group and the normal group, we then collected all sequenced genomes and estimated the probabilities that different strains may exist in the samples through an unbiased sequence reassignment algorithm (Fig. 1A). In brief, the first step was to screen out species with strain heterogeneity in the disease group and the healthy group; and the second step was to explore the details of the target species’ strain heterogeneity and relationship with the disease.

10.1128/mSystems.00775-21.6 FIG S1 Pipeline of the single-nucleotide polymorphism (SNP)-calling procedure. Download FIG S1, TIF file, 0.03 MB. Copyright © 2021 Chen et al. 2021 Chen et al. https://creativecommons.org/licenses/by/4.0/ This content is distributed under the terms of the Creative Commons Attribution 4.0 International license.

We investigated the SNP patterns of microbes in the LC and HC samples. First, we selected 13 representative strains from different species with >40% genome coverage and >10× sequencing depths in >20 samples per group (see Table S1 in the supplemental material). The selected thresholds were determined according to an influential study on microbial SNPs published by Schloissnig et al. in 2012 (10). Based on the genomes of these 13 strains, each strain being the reference genome for its corresponding species, we detected a total of 3.94 million high-quality SNPs. The SNP density distribution of *F. prausnitzii* (reference strain KLE1255, GenBank accession no. GCA_000166035.1) alone differed significantly between groups (*P = *4.7 × 10^−7^; *q *= 6.5 × 10^−6^) (Fig. 1B). The SNP density differentiation of *F*. *prausnitzii* suggested that its strain compositions may differ between HC and LC groups.

10.1128/mSystems.00775-21.2 TABLE S1 Detailed descriptive information for the 13 selected bacterial strains. Download Table S1, XLSX file, 0.01 MB. Copyright © 2021 Chen et al. 2021 Chen et al. https://creativecommons.org/licenses/by/4.0/ This content is distributed under the terms of the Creative Commons Attribution 4.0 International license.

Then we constructed a read reassignment-based pipeline and examined the strain diversity of gut microbes. Since *F. prausnitzii* was indicated as a species with significantly different strain compositions in the LC and HC groups, we focused on *F. prausnitzii* as the target in our following analysis. In order to evaluate the reliability of the sequence reassignment algorithm, we generated simulated metagenomic sequencing data to test the performance of our pipeline. In total, 136 assembled *F. prausnitzii* genomes were collected from National Center for Biotechnology Information (NCBI) (see Table S2A in the supplemental material) (26). We sought to simulate the scenario that a random number (*n* = 1 to 10) of *F*. *prausnitzii* strains were present in one sample, together with the presence of 1 to 100 other species that were also detected in real gut metagenomic samples. Since we knew the real community composition of the synthetic samples, we compared the estimated strain profiles from our pipeline to the actual profiles; the estimated values (coverages, depths, and abundances) and the actual values showed a correlation of >0.92, indicating that our pipeline performed reliably (Fig. 1C). The scatterplot of correlation coefficients shows that the estimated coverage values were generally slightly larger than the actual values (Fig. 1C, top), as coexisting similar genomes could contribute to each other’s read coverage. However, we believe that this minor bias should not be considered erroneous, but as inherent information provided by the data, since highly similar coexisting strains increase the probability of each other’s recognition.

10.1128/mSystems.00775-21.3 TABLE S2 (A) The 136 Faecalibacterium prausnitzii genomes. (B) Clustering results of the 136 F. prausnitzii strains. Download Table S2, XLSX file, 0.03 MB. Copyright © 2021 Chen et al. 2021 Chen et al. https://creativecommons.org/licenses/by/4.0/ This content is distributed under the terms of the Creative Commons Attribution 4.0 International license.

It should be noted that characteristics of a strain estimated by our pipeline, such as the estimated coverages, depths, and abundances are more like indicators of the probabilities of strains being present in the sample. To take the genome coverage as an example, the higher the estimated coverage, the greater the probability that the strain exists in the sample. In actual situations, when we do not know the strain composition of a sample in advance, we can make predictions about the probabilities that known strains are present in the sample; we can also infer the similarities between the actual strains in the sample and the known strains in the database according to the corresponding estimated values like coverages, depths, and abundances.

### Different strain profiles of *F. prausnitzii* in the LC and HC group.

**FIG 2:**
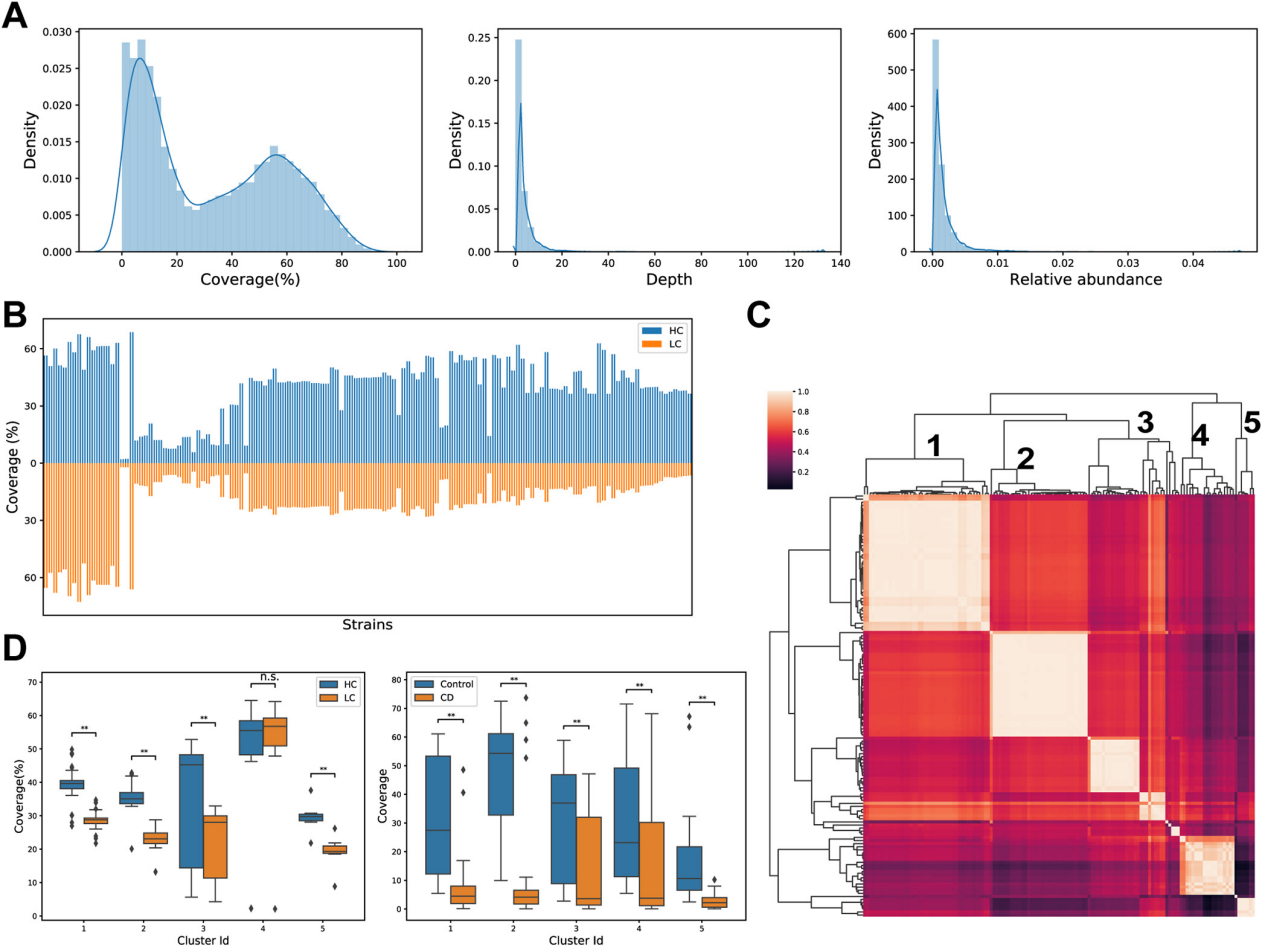
Heterogeneity of *F. prausnitzii* strains in the disease and healthy groups. (A) The distributions of estimated coverages, depths, and relative abundances of the 136 *F. prausnitzii* strains in real samples. (B) Estimated read coverage for the 136 *F. prausnitzii* strains. (C) Clustering of *F. prausnitzii* strains according to their prevalence in samples. (D) Estimated coverage distributions of strain clusters in the healthy control and liver cirrhosis groups (left) and in the healthy control and Crohn’s disease groups (right).

We used our pipeline to infer the existence probabilities of the 136 *F. prausnitzii* strains in the LC and HC groups. Figure 2A shows the distributions of estimated coverages, depths, and abundances of *F. prausnitzii* strains in the cohort. Estimated coverage values in the samples showed obvious bimodal distribution compared to those of depths and abundances, indicating the possible heterogeneity reflected by genome coverages. Thus, we selected estimated coverages of strains as the targets of subsequent analysis. Figure 2B shows the comparison of average coverages of these strains between the two groups, which also indicates an obvious heterogeneity in the existence of the strains in the two groups. We grouped the genomes into clusters according to the coverages, which is highly consistent with the clustering result based on core gene sequences (adjusted Rand index = 0.84; see Fig. S3 in the supplemental material). The genomes were grouped clearly into at least five clusters (Fig. 2C and Table S2B). The cluster 4 (C4) strains showed similar coverages in HC samples as in LC samples, whereas the other four clusters of strains showed far lower coverages in the LC group than in the HC group, which is consistent with the results at species level reported previously by other researchers (Fig. 2D, left).

10.1128/mSystems.00775-21.8 FIG S3 A phylogenetic tree constructed using conserved genes from *F. prausnitzii* strains. Coverage-based clustering results in our paper are indicated by the last two characters of node names and by different background colors. According to the figure, the two clustering results, genome-based versus coverage-based, achieved a high degree of consistency. See Materials and Methods in the main text for conserved gene identification in the *F. prausnitzii* strains. Genomes without available GFF annotation were excluded from the phylogenetic tree construction. To cluster the strains based on the tree, TreeCluster software was used. Download FIG S3, TIF file, 0.3 MB. Copyright © 2021 Chen et al. 2021 Chen et al. https://creativecommons.org/licenses/by/4.0/ This content is distributed under the terms of the Creative Commons Attribution 4.0 International license.

The diversity of *F. prausnitzii* genomes was also noticed in earlier studies (19, 22, 24). However, only 17 to 34 sequenced *F. prausnitzii* genomes were involved in those studies, and the strains were grouped into two clusters, phylogroup I and phylogroup II. We used the most comprehensive *F. prausnitzii* genomes (136 genomes) in our study, which permitted an overall perspective of strain diversity of *F. prausnitzii*. The strains in phylogroup I were all grouped into cluster 1 in our study, whereas strains in phylogroup II were more finely grouped into different clusters in our study (see Table S3 in the supplemental material). Our clustering results may reveal the most comprehensive diversity of *F. prausnitzii* strains related to LC known so far.

10.1128/mSystems.00775-21.4 TABLE S3 Clustering result of *F. prausnitzii* strains in different studies. Download Table S3, XLSX file, 0.01 MB. Copyright © 2021 Chen et al. 2021 Chen et al. https://creativecommons.org/licenses/by/4.0/ This content is distributed under the terms of the Creative Commons Attribution 4.0 International license.

We also analyzed metagenomic data from a new cohort that included patients with Crohn’s disease (CD) and healthy individuals (27). We found that, unlike in LC, the C4 strains were significantly less abundant in CD samples relative to those from healthy individuals (*P = *2.2 × 10^−4^, Mann-Whitney test; Fig. 2D, right). This result suggests that the C4 strains may perform different functions in intestinal microenvironments in LC patients and in CD patients.

### Functional differences of *F. prausnitzii* among different clusters.

**FIG 3:**
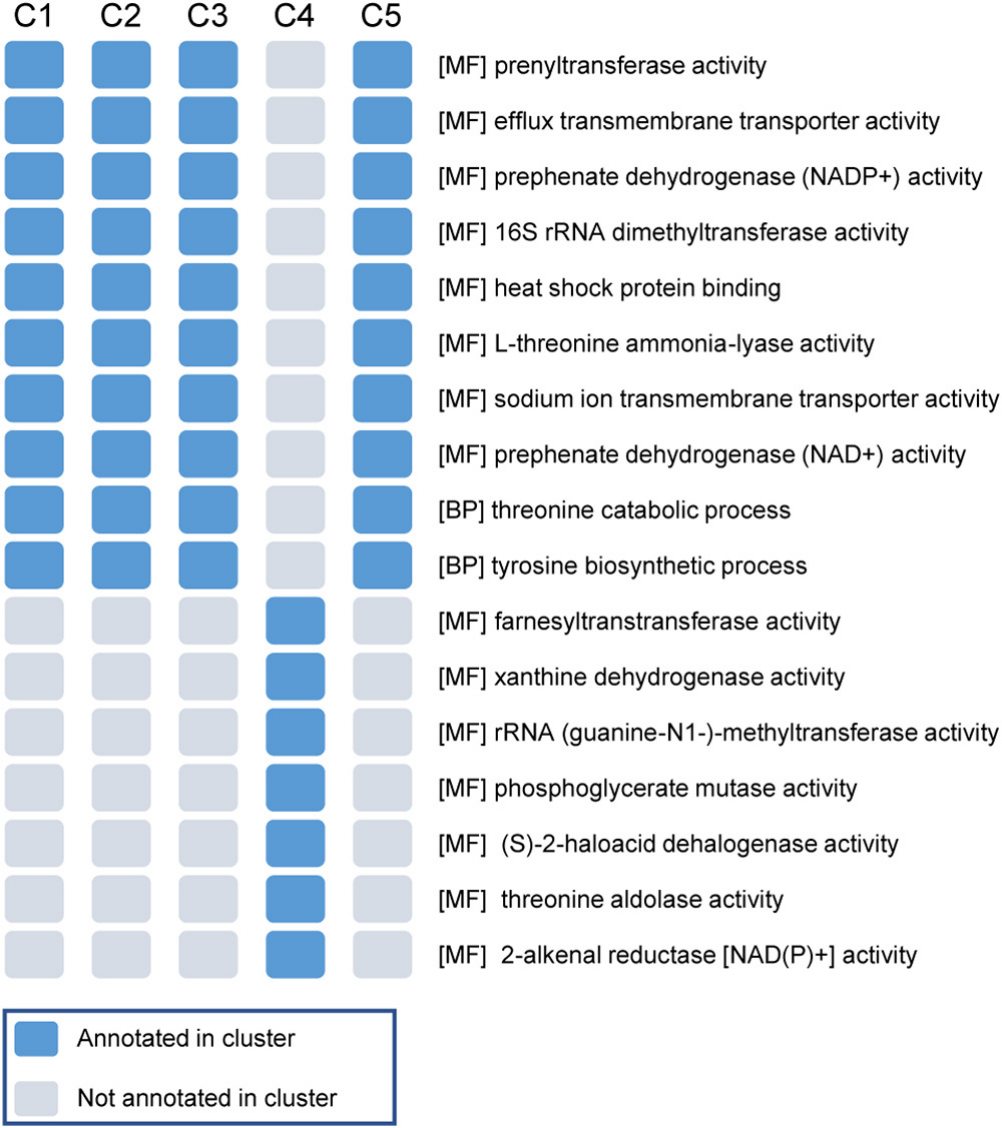
Gene Ontology (GO) terms deficient and specific for cluster 4 (C4) strains. The blue square indicates that the corresponding GO term on the right is annotated in the cluster.

We annotated the protein sequences of the 136 *F. prausnitzii* strains to UniRef90 and Gene Ontology (GO) terms using HMP Unified Metabolic Analysis Network (HUMAnN) data files. Since the C4 cluster was the most special subgroup in clustering results, we obtained GO terms that were only annotated in genomes of the C4 cluster and not in genomes of the other clusters (Fisher’s exact test, *P* = 1.32 × 10^−23^). GO terms annotated for strains in other clusters but not for C4 strains were also obtained (Fig. 3). Khan and colleagues (28) reported that *F. prausnitzii* strain A2-165 and HTF-F, which are in different clusters according to our results, showed different short-chain fatty acid (SCFA) production efficiencies under oxygenated growth conditions and anoxic conditions in the presence of fumarate, in which the transformation of NADH to NAD^+^ and extracellular electron transfer played important roles. In our results, several activities related to NAD^+^ and the transmembrane transporter were identified that contributed to the functional differences between the C4 cluster and other clusters of *F. prausnitzii* strains, implying that the differences in functions between the C4 cluster and the other clusters may involve SCFA production processes.

We also examined Kyoto Encyclopedia of Genes and Genomes (KEGG) pathway differences among *F. prausnitzii* genomes in different clusters. We found that the pathways of propanoate metabolism, arginine biosynthesis, and d-glutamine and d-glutamate metabolism were not annotated in the cluster 2 (C2) genomes (Fisher’s exact test, *P* = 7.76 × 10^−33^). The chloroalkane and chloroalkene degradation (mainly 2-haloacid dehalogenase), chlorocyclohexane and chlorobenzene degradation (also 2-haloacid dehalogenase), and RNA transport (mainly RNase Z) pathways were annotated only in the C4 genomes.

We then compared copies of the conserved *F. prausnitzii* genes identified among strains in different clusters. Genes with the fewest differences between average intercluster and intracluster distances, which are more conserved among clusters in sequence and functional perspectives, mainly encode 50S ribosomal protein, 30S ribosomal protein, and translation initiation factor IF-1 (see Table S4 in the supplemental material). Genes with the most differences, which are less conserved or more specific to individual clusters, included those encoding several proteins annotated as integral membrane components (GO no. 0016021), such as FeoB-associated Cys-rich membrane protein. A TrkA-family potassium uptake protein with diverse sequences among clusters was also detected. This protein can bind to NAD^+^ and NADH, according to UniProtKB (29), and is involved in potassium ion transmembrane transporter activity (GO no. 0015079); this finding was consistent with our GO annotation results. We also observed that several conserved genes related to membrane proteins were more conserved (nearly identical) in the C4 strains but more diverse in strains in other clusters. These results suggest the existence of physiological differences among the *F. prausnitzii* clusters. The associations of distinct strains with different pathways may also shed light on studies of the association between *F. prausnitzii* and LC.

10.1128/mSystems.00775-21.5 TABLE S4 Average intracluster and intercluster distances of conserved genes. Download Table S4, XLSX file, 0.02 MB. Copyright © 2021 Chen et al. 2021 Chen et al. https://creativecommons.org/licenses/by/4.0/ This content is distributed under the terms of the Creative Commons Attribution 4.0 International license.

### Ability of *F. prausnitzii* strains to discriminate between LC and HC samples.

**FIG 4:**
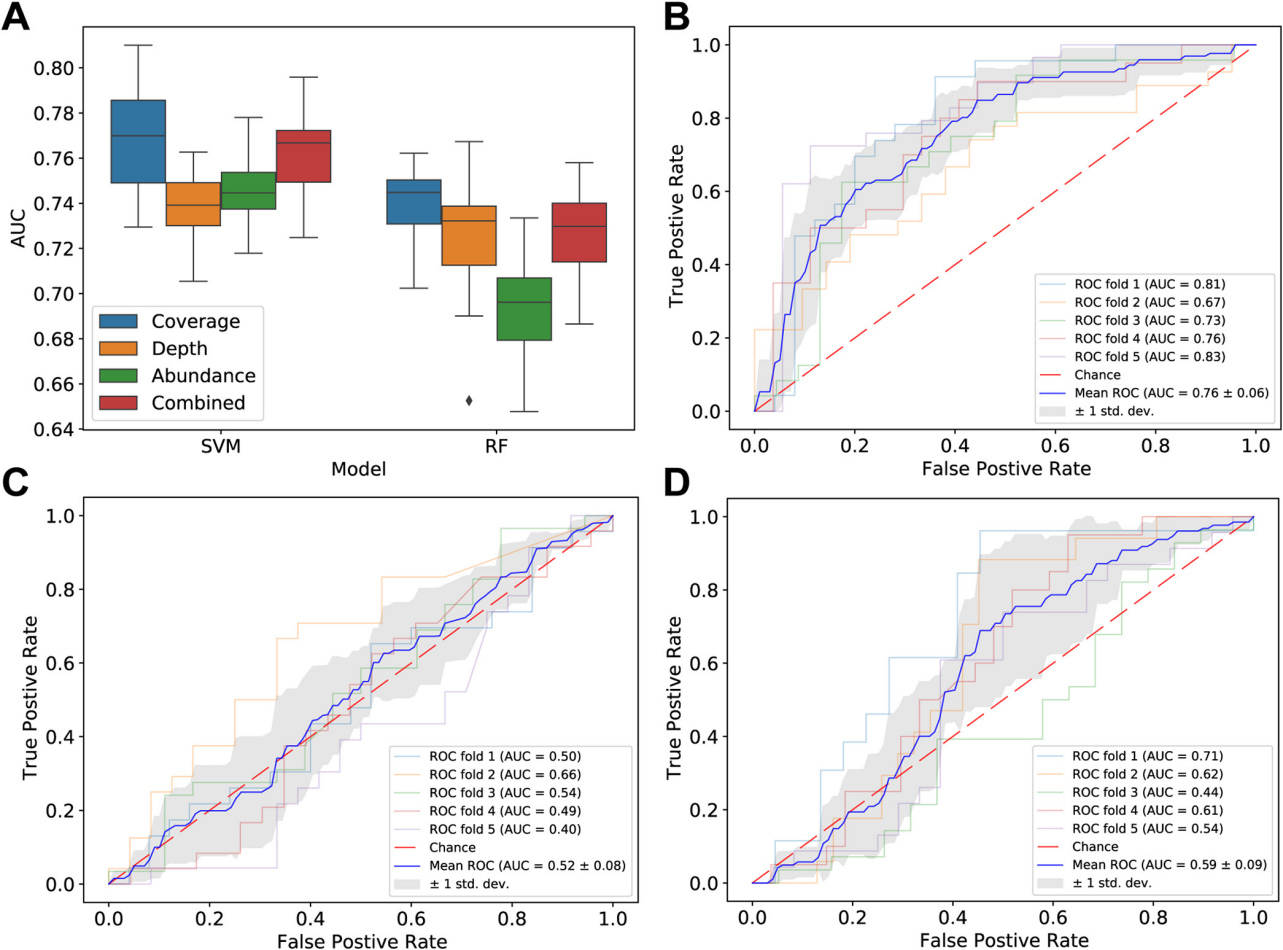
Performances of machine learning models to distinguish disease states. (A) Performances of different combinations of strain-level data and machine learning models. “Combined” represents the combination of coverage, depth, and abundance. (B) Receiver operating characteristic (ROC) curves for support vector machine (SVM) models to classify samples using estimated coverages of GCA_001406615.2 in samples. (C) ROC curves for SVM models to classify samples using estimated coverages of GCA_902388275.1 in samples. (D) ROC curves for SVM models to classify samples using abundances of *F. prausnitzii* species from MetaPhlAn2 results.

To determine whether the HC and the LC samples could be discriminated based on strain features estimated by our pipeline, we trained machine learning models with different combinations of features (coverages, depths, and abundances) as the input and sample status (LC or HC) as the output. The results show that whether using support vector machine (SVM) or random forest (RF) models, taking coverage as input alone can produce the best prediction performances (Fig. 4A). The coverage-based SVM models could achieve a median area under the receiver operating characteristic curve (AUC) of 0.77, higher than those obtained with other data-model combinations. Random forest models revealed that the most important feature for the prediction performances was the estimated coverage of GenBank accession number GCA_001406615.2 (C5 strain 2789STDY5834930). We then used only the estimated coverage of GCA_001406615.2, rather than coverages of all 136 strains, to model the disease states and achieved a comparable performance (SVM AUC = 0.76, Fig. 4B; RF AUC = 0.72; Fig. S4A). This result shows the impressive ability to use a single strain of *F. prausnitzii* as the reference to distinguish LC and HC samples. Replacement of the GCA_001406615.2 genome with that of another strain, such as GenBank accession number GCA_902388275.1, reduced the discriminatory performance (SVM AUC = 0.52, Fig. 4C; RF AUC = 0.52, Fig. S4B), demonstrating the heterogeneity of *F. prausnitzii* strains. It is of note that we checked the effects of confounder factors (age, sex, and body mass index [BMI]) and confirmed that the confounders have little effect on estimated coverages of *F. prausnitzii* strains in real samples (see Fig. S5 and Text S1, “Confounder analysis,” in the supplemental material).

10.1128/mSystems.00775-21.1 TEXT S1 Supplementary explanations of the methods in the main text. Sections include “Confounder analysis,” “Comparisons between species-level and strain-level data,” and “PERMANOVA analysis.” Text S1, DOCX file, 0.02 MB Copyright © 2021 Chen et al. 2021 Chen et al. https://creativecommons.org/licenses/by/4.0/ This content is distributed under the terms of the Creative Commons Attribution 4.0 International license.

10.1128/mSystems.00775-21.9 FIG S4 Performances of machine learning models to distinguish disease states. (A) Receiver operating characteristic (ROC) curves for random forest models to classify samples using estimated coverages of GCA_001406615.2 in samples. (B) ROC curves for random forest models to classify samples using estimated coverages of GCA_902388275.1 in samples. (C) ROC curves for random forest models to classify samples using *F. prausnitzii* species abundances from MetaPhlAn2 results. (D) Using age-balanced datasets, performances of models based on strain coverages are better than that of models based on MetaPhlAn2-derived abundances. The age-balanced dataset is explained in the “Confounder analysis” section in Text S1 in the supplemental material. Download FIG S4, TIF file, 0.3 MB. Copyright © 2021 Chen et al. 2021 Chen et al. https://creativecommons.org/licenses/by/4.0/ This content is distributed under the terms of the Creative Commons Attribution 4.0 International license.

10.1128/mSystems.00775-21.10 FIG S5 Confounder effect analysis. Variables biases for age (A), body mass index (BMI) (B), and sex (C) in the two groups. (D) Correlation between ages and estimated strain coverages. (E) Model performances on the age-balanced subsets. Download FIG S5, TIF file, 0.2 MB. Copyright © 2021 Chen et al. 2021 Chen et al. https://creativecommons.org/licenses/by/4.0/ This content is distributed under the terms of the Creative Commons Attribution 4.0 International license.

To illustrate the necessity of strain-level resolution, we compared the *F. prausnitzii* species abundances given by MetaPhlAn2 with the strain coverages given by our process. Permutational multivariate analysis of variance (PERMANOVA) showed that the strain-level results can better characterize the beta-diversity of the LC group and the HC group (Text S1, “PERMANOVA analysis”). When using the results of MetaPhlAn2 for disease state modeling, the performance of the model is much lower than that of the strain-level models (SVM AUC = 0.59 [Fig. 4D]; RF AUC = 0.54 [Fig. S4C]; see also Text S1, “Comparisons between species-level and strain-level data”). These results indicate the species-level data’s insensitivity to the prediction of disease state.

It should be noted that after our analysis was completed, we found that GCA_001406615.2 was marked as “Anomalous assembly” and was excluded from the RefSeq database, but still remained in the GenBank database. Therefore we re-performed the analysis after excluding the genome of this strain. We used the estimated coverages of the remaining 135 strain genomes to model the disease states, which can achieve comparable results (mean SVM AUC = 0.73, mean RF AUC = 0.72); similarly, we found a strain with the best modeling performance (GCA_002549905.1, estimated depths as inputs, SVM AUC = 0.76, RF AUC = 0.74), which is included in both the GenBank and RefSeq databases. These results are consistent with the previous conclusions, including the confounder analysis part. However, since GCA_001406615.2 belongs to the cluster 5 in our analysis, researchers may need to pay attention to the potential abnormalities of the genomes of other strains in the cluster 5; on the other hand, considering the genomes of the strains from one cluster are highly similar, we also need to discuss whether the so-called assembly abnormalities of these strains come from contamination or actually from genome integrations like the horizontal gene transfer of the microbial community.

## DISCUSSION

This work provides a computational framework of strain-level analysis in gut metagenomes and reports a systematic examination of *F. prausnitzii* strain diversity in relation to LC. Our results suggest that the strains in the same species may exert different functions, and certain strains, rather than the whole species, likely provide useful information for LC diagnosis and treatment. Strain heterogeneity may have been overlooked in previous metagenomic studies.

SCFAs are considered to be important for interactions between beneficial microorganisms and hosts, and *F. prausnitzii* is considered to be among the main bacterial SCFA producers. Based on our annotations of the functional pathways of different strain genomes, we conclude that SCFA metabolism may differ among strain clusters. In addition, the microbes in the C4 cluster were related specifically to the metabolic pathways of chlorine-related compounds, which may be associated with the lack of difference in their abundance between the LC and HC groups. However, in contrast to the LC/HC results, the C4 strains showed reduced abundance in patients with CD relative to those in HCs. These findings indicate that the C4 strains might perform different functions in different diseases. Furthermore, the functional differences among the other clusters also demonstrate the potentially diverse roles that different strains play in human health.

More experiments need to be conducted so as to confirm the hypothesis of the physiological differences among subgroups of *F. prausnitzii* strains. What needs to be pointed out is that our estimated coverages of strains can only indicate the present probabilities of corresponding strains in samples or the similarities between actual strains in samples and known strains or clusters in databases. Nonetheless, our results suggest that strain heterogeneity should receive more attention. Recently developed single-cell microbial sequencing technologies seems to be more promising for metagenomic analysis (30, 31), especially at the strain level. With the rapid development of sequencing technologies and experimental approaches, an increasing number of metagenomic studies will involve strain-level analysis. Such analysis of human metagenomes can help researchers develop more reliable disease diagnosis and treatment methods (e.g., probiotic use and safe microbiota transplantation) from a microbiological perspective.

## MATERIALS AND METHODS

### Data sources.

Raw sequencing data sets of DNA extracted from fecal samples from 123 Chinese patients with LC and 114 Chinese HCs were downloaded from the National Center for Biotechnology Information (NCBI) database (accession no. ERP005860) (32). The overall data set comprised about 566 Gb, with an average of 2.4 Gb per sample. We also downloaded partial data sets comprising 25 samples from patients with CD and 17 samples from HCs from the NCBI Sequence Read Archive (accession no. SRP129027) (27). These data covered 162 Gb, with an average of 3.9 Gb per sample.

### Microbial SNP calling.

We called microbial SNPs using a computational framework employed previously (33). Briefly, we first performed quality control on raw data and then used MetaPhlAn2 (34) to profile the microbial compositions in samples. Species detected in more than three samples were reserved as the final reference set, and one reference strain was selected as a reference for each species. Then, we filtered the strains by mapping reads to the reference with the Burrows-Wheeler Aligner (35) and retained only strain genomes with sufficient reads (>40%) and sequencing depths (>10×) covered in at least 20 samples in each of the respective HC and LC groups. SAMTools (36) was used to call SNPs with the parameters “-vmO z -V indels,” and the results were filtered using VCFTools with the parameters “+/d = 10/a = 4/Q = 15/q = 10/.” To reduce the number of false-positive results, VarScan2 (37) was also used to call SNPs with the parameters “--min-coverage 10 --min-reads2 4 --min-var-freq 0.2 --p-value 0.05.” SNPs detected by both SAMTools and VarScan2 were selected for the next step of the analysis.

### Downsampling.

We compared the distributions of read counts in samples between the HC and LC groups. The two groups had similar read count distributions, except that several more deeply sequenced samples from the LC group had read counts exceeding 40 million. We randomly downsampled these outlier samples to the mean populational read count to make the two sets of samples consistent in size distribution (Mann-Whitney test, *P* = 0.002 before downsampling; *P* = 0.08 after downsampling; see Fig. S2 in the supplemental material).

10.1128/mSystems.00775-21.7 FIG S2 Read count distributions in the healthy control (HC) and liver cirrhosis (LC) groups. (Left) Raw read count distribution of samples. (Right) Read count contribution of the downsampled samples. Download FIG S2, TIF file, 0.1 MB. Copyright © 2021 Chen et al. 2021 Chen et al. https://creativecommons.org/licenses/by/4.0/ This content is distributed under the terms of the Creative Commons Attribution 4.0 International license.

### Strain diversity inference.

To rigorously infer the strain diversity of *F*. *prausnitzii* in metagenomic samples, we first removed reads from other bacterial genomes using the reference genome set that we built using MetaPhlAn2. We mapped the total reads against this background reference set using Bowtie 2 (51); reads that mapped to any background genome were discarded. We downloaded a total of 136 assembled *F*. *prausnitzii* genomes from the NCBI genome database (26). Two mapping steps were implemented to assign reads more reliably to their genomes of origin. First, the reads were mapped to the reference collection of 136 *F. prausnitzii* genomes in competitive mode to identify those that mapped best to single genomes. To avoid noise and bias induced by genome mixture and the alignment tools, we then aligned the reads to each *F. prausnitzii* genome separately in exclusive mode. All reads that mapped to a given genome (*G*) were considered to be candidate reads assigned to *G*. Reads that mapped best only to *G* (type 1 [T1]) were retained, those that mapped best to other genomes (type 2 [T2]) were discarded, and reads that mapped simultaneously to *G* and to other genomes (type 3 [T3]) were assigned conditionally to those multiple genomes. A T3 read aligned to *G* with mismatches was not assigned to *G* if it overlapped with T1 reads with fewer mismatches; in all other cases, T3 reads were assigned to *G*. Alignments with ≥5 mismatches per 100 bp were not considered to be valid.

### Simulation of the *in silico* community.

To test the performance of the strain diversity pipeline, we simulated metagenomic samples *in silico*. We used the modified reference set (without *F. prausnitzii*) as a background genome set and mixed it with subsets of the 136 *F. prausnitzii* genomes to generate the simulated samples. For each sample, 1 to 10 *F. prausnitzii* genomes and 1 to 100 background genomes were selected randomly. The read fraction of each genome was also determined randomly, with all read fractions summing to 1. The read length was set to 100 bp. For each read, a mutation mechanism was also introduced, and a maximum of five substitutions was allowed.

### Support vector machine and random forest model training.

The Python package *scikit-learn* (38) was used to train both support vector machine (SVM) and random forest (RF) models. For both models, a randomized search of hyperparameter and 5-fold cross-validation strategy was utilized to achieve the best performances. For SVM models, parameter C, gamma, kernel, and class weight were searched; for RF models, estimator numbers, maximum depth, maximum features, maximum leaf nodes, minimum sample split, and bootstrap or not were searched.

### Conserved gene identification and phylogenetic tree building.

We collected coding DNA sequences (CDSs) from the RefSeq GFF files (39) for the *F. prausnitzii* strains. The M21/2 strain CDS was taken as a reference for the alignment of CDSs from all other strains using *parasail-python* (40). Pairwise alignment scores for two sequences were normalized using the self-aligned scores of each sequence. When a similar copy of one reference CDS (score > 0.5) was detected in all available strains, this CDS was designated conserved. Clustal Omega was used for multiple-sequence alignment (41) and RAxML (42) version 8 was used for phylogenetic tree building. Clustering using TreeCluster (43) was performed based on the phylogenetic tree.

### Functional analysis.

We used the data files from HMP Unified Metabolic Analysis Network (HUMAnN) version 3.0 (44) to annotate the functions of *F. prausnitzii* genomes. Diamond (45) was used to align the *F. prausnitzii* protein sequences to the HUMAnN-derived UniRef90 database (46). The relationships among UniRef90, Gene Ontology (GO) (47, 48), and Kyoto Encyclopedia of Genes and Genomes (KEGG) orthology (KO) (49) terms were also determined using the HUMAnN data files. Thus, each protein sequence was annotated with UniRef90, GO, and KO terms. Then, we used KEGG Mapper (52) to reconstruct genome pathways.

### Statistical analysis and other software utilizations.

The Mann-Whitney test was used to identify differences in strain relative abundance and SNP densities between the LC and HC groups. The R package *qvalue* (version 2.10.0) (50) was used to control the false-discovery rate. Fisher’s exact test was used to measure the significance of the functional difference among different clusters. PERMANOVA was performed using the *skibio* (http://scikit-bio.org) package. Adjusted Rand index was calculated using *Scikit-learn* (38). The Python package *statsmodel* (53) was used to perform the confounder analysis.

### Source code availability.

The source code of our pipeline and related Jupyter notebooks have been posted on GitHub (https://github.com/labomics/; “metagenomic_SNP_calling” and “strain_profiling” projects).
