# Supplementary figures and images for: Comprehensive Strain-Level Analysis of the Gut Microbe Faecalibacterium prausnitzii in Patients with Liver Cirrhosis
Source: mSystems. 2021 Aug 3;6(4):e00775-21. doi: 10.1128/mSystems.00775-21 (PMC8407477; doi:10.1128/mSystems.00775-21)

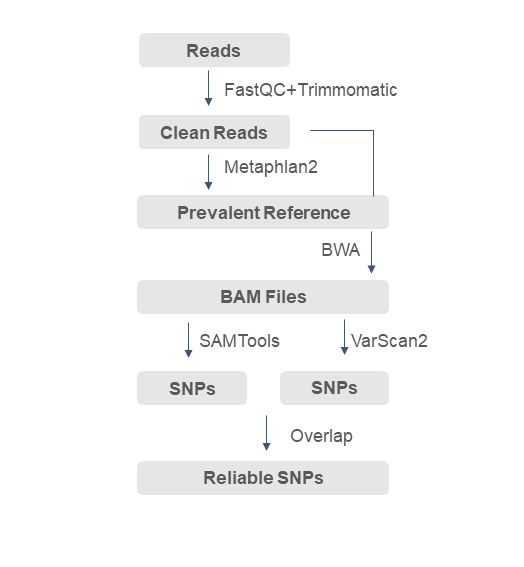

Supplement: FIG S1 [file msystems.00775-21-sf001.tif]

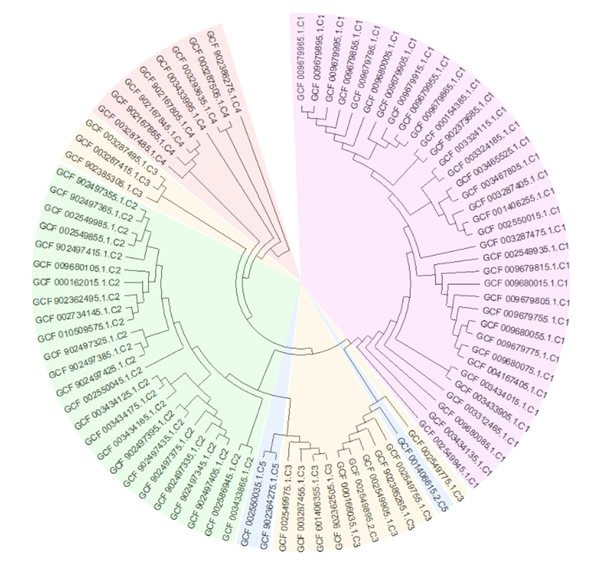

Supplement: FIG S3 [file msystems.00775-21-sf003.tif]

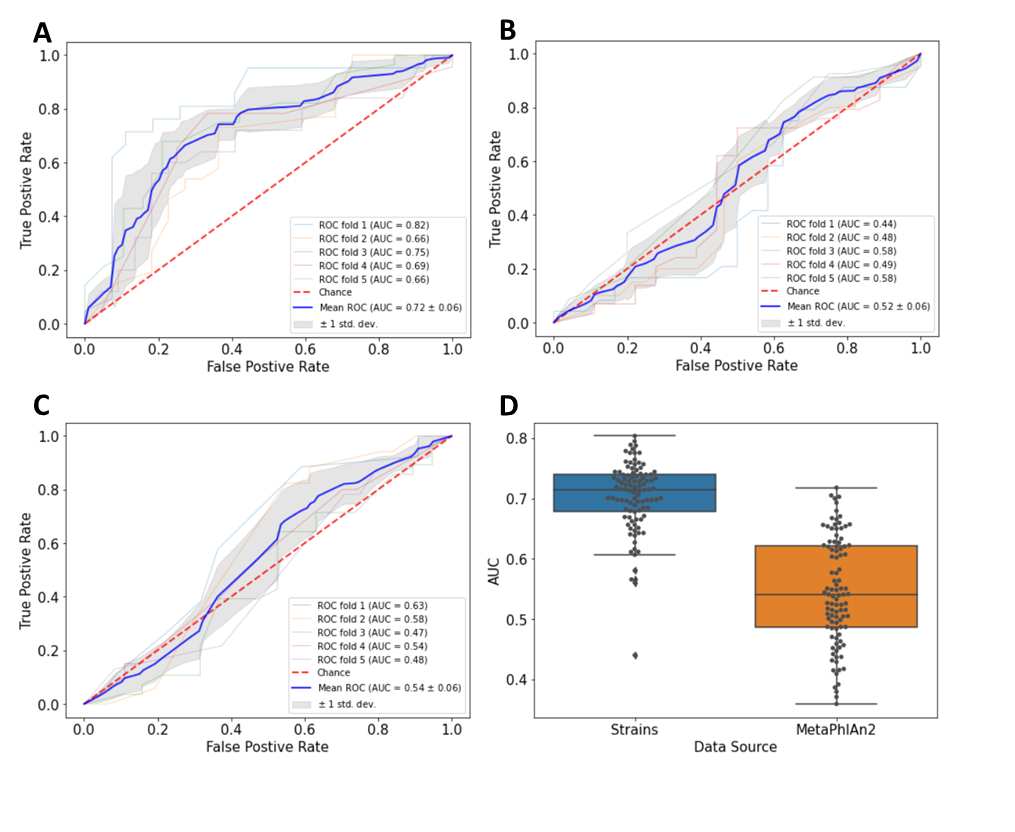

Supplement: FIG S4 [file msystems.00775-21-sf004.tif]

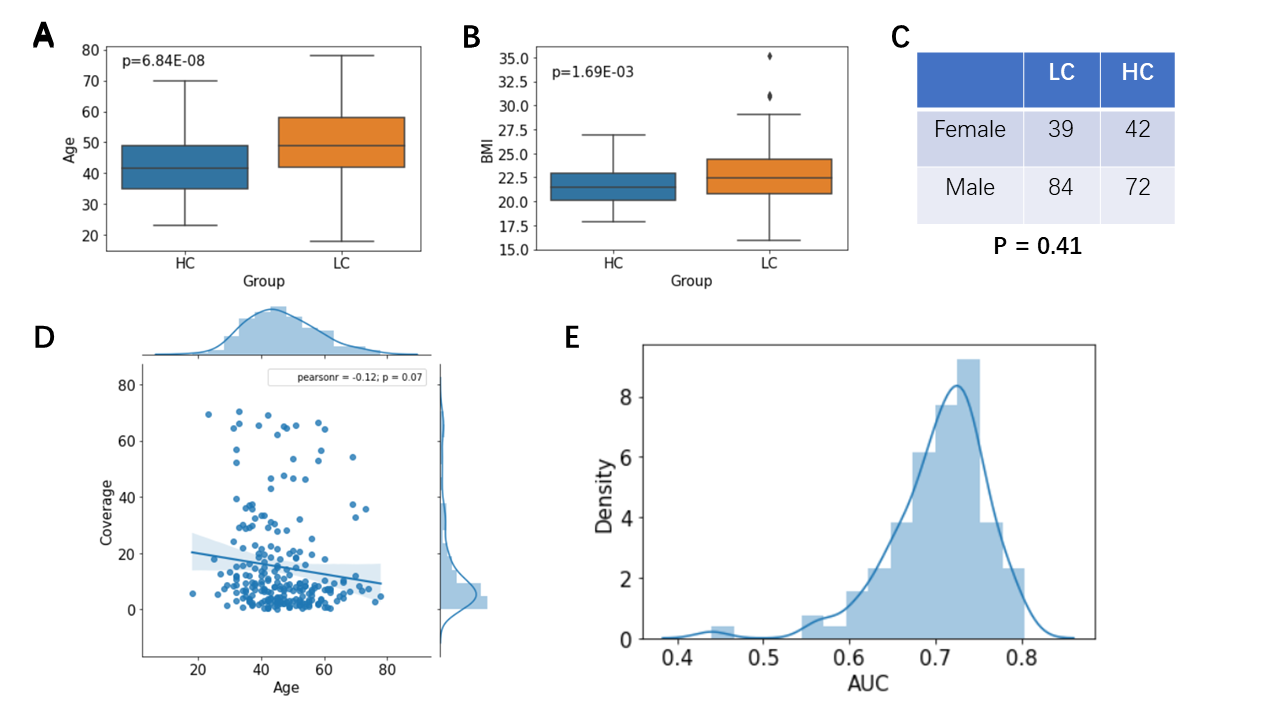

Supplement: FIG S5 [file msystems.00775-21-sf005.tif]

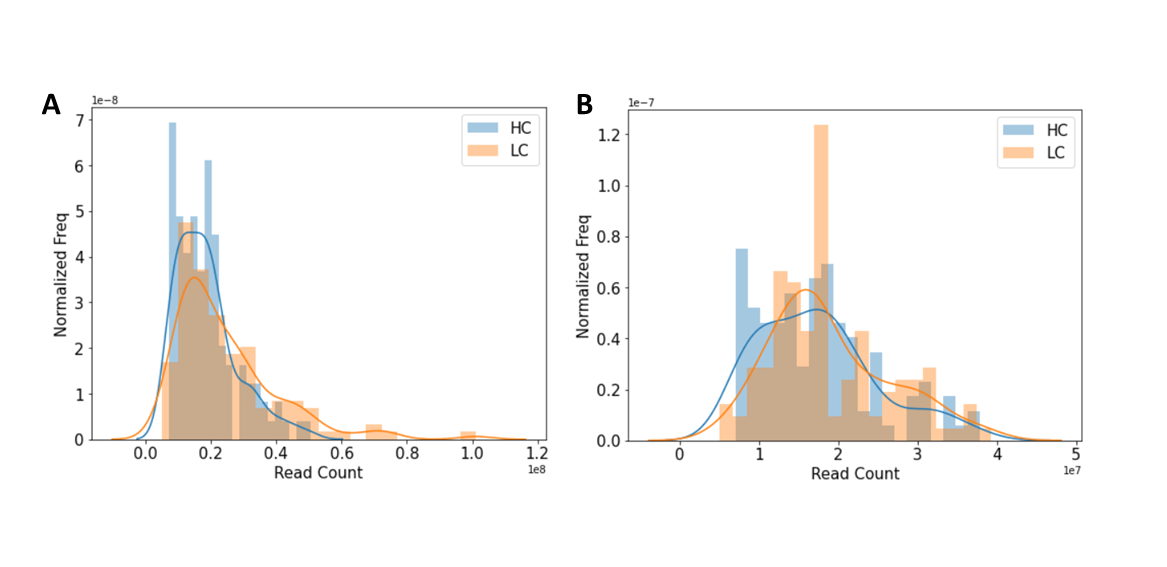

Supplement: FIG S2 [file msystems.00775-21-sf002.tif]
