# Supplementary material for: Comprehensive Strain-Level Analysis of the Gut Microbe Faecalibacterium prausnitzii in Patients with Liver Cirrhosis
Source: mSystems. 2021 Aug 3;6(4):e00775-21. doi: 10.1128/mSystems.00775-21 (PMC8407477; doi:10.1128/mSystems.00775-21)
Supplement: TEXT S1 [file msystems.00775-21-t0001.docx]

**Confounder effect analysis**

Since the meta information of samples includes gender, age, and BMI, we first checked whether there are differences in the distributions of these three variables between the LC and HC groups. The results showed that there was a statistical bias in ages between LC and HC samples (Mann Whitney test, p = 6.8e-08, Figure S5A); BMIs were also biased in the two groups (Mann Whitney test, p = 0.002, Figure S5B); Gender bias did not exist in the two groups (Fisher's exact test, p = 0.41, Figure S5C).

We tried to use these variables to predict disease status (SVM, 5-fold cross-validation), and found that ages could predict disease status to a certain extent (average AUC 0.68), while BMIs and genders cannot predict disease status (AUC 0.52 and 0.53, respectively). The results showed that ages are biased in the current grouping. In order to check whether the estimated strain coverage profiles of samples from our pipeline are related to sample ages, we calculated the correlation between ages and estimated coverages of GCA_001406615.2, which is the most important feature in our random forest model. The results showed that the correlation between these two variables is not significant (R = -0.12, p = 0.07, Figure S5D), leading to the assumption that the estimated coverages of strains in samples and the ages of patients can independently distinguish disease states.

Next, we used an ordinary least-squares (OLS) model from the python package *statsmodels* to perform linear regression fitting to the disease state taking estimated strain coverages, patient ages, sexes, and BMIs as inputs. The results showed that coverages and ages have significant coefficients (coverage: -0.096, age: 0.0129, both p-values<<0.05); when we considered the interaction between coverages and ages, the interaction term *coverage*age* got a coefficient of 8.87e-06 and a p-value of 0.952, which indicates that there is no interaction between the two variables. The above results show that ages of patients and strain coverages could independently predict disease status. When taking both the strain coverages and ages as inputs to the model, its performance to distinguish between diseases and normal states (AUC 0.77) is higher than that of using two variables alone (AUC 0.76 and 0.68 for coverages and ages, respectively).

Next, we randomly sampled the data set to get a balanced subset that there is no significant difference in ages between the LC and HC groups (Mann Whitney test, p>0.1). We performed 100 times of samplings that met the requirements. In each case, we used estimated coverages of GCA_001406615.2 to predict the disease states of the two groups, and the final median AUC value is above 0.7 (Figure S5E). The results showed that the estimated coverages of *F. prausnitzii* strains in samples were independent of ages, genders etc. and could be used as indicators to distinguish between the LC and HC samples.

**Comparisons between species-level and strain-level data**

Since we only used the strain composition of *F. prausnitzii* to distinguish disease states, we used the estimated abundances of *F. prausnitzii* by MetaPhlAn2 to distinguish disease states. Although the abundances of *F. prausnitzii* estimated by MetaPhlAn2 in the HC group was significantly higher than that in the LC group, the AUC of the SVM model to distinguish disease states constructed based on MetaPhlAn2 results was only 0.59 (RF AUC 0.52, Figure 4D, S4C).

In order to exclude the influence of age on grouping, we generated multiple sample subsets to keep the age balanced in the grouping of the subsets. The results show that our model can get a media AUC of 0.71, while that of MetaPhlAn2-based models is 0.54 (Figure S4D). It shows that our method is more effective after age correction.

We also considered the reasons for the poor performances of MetaPhlAn2 results. We think that for *F. prausnitzii*, when MetaPhlAn2 constructs the marker set, multiple genome markers are mixed, including GCA_000210735.1 from the cluster 4, GCA_000166035.1 from the cluster 3, GCA_000154385.1, GCA_000209855.1 from the cluster 1, and GCA_000162015.1 from the cluster 2. These mixed strain markers make the method less sensitive, considering that different strain clusters have different distributions in the disease group and the control group. Although *F. prausnitzii* species was found significantly reduced in relative abundance in the LC group, its ability to distinguish disease is still not strong enough.

It should be pointed out that we only use the relative abundance values of *F. prausnitzii* here. If the abundance values of all species were used, the model performance would be better. Since our focus is on the strain heterogeneity of *F. prausnitzii* in the LC and HC groups, the ability of the strain profile to distinguish diseases was used to confirm the actual difference in the distribution of strains between the two groups. Of course, it is very important and significant to consider the profile of all strains, not just *F. prausnitzii*, to model the disease state, and we will continue to study it in the follow-up work.

**PERMANOVA analysis**

We performed a PERMANOVA analysis to distinguish between the healthy group and the disease group. Still, we only considered *F. prausnitzii* species here.

We used the PERMANOVA method from the Python package *skibio* to perform the beta-diversity analysis. Using estimated coverages as input, permuting 100,000 times to test whether there is a difference between the microbial communities in the LC and HC groups, the test statistic was 40.0679, and the p value was 1e-05; but using results from MetaPhlAn2 as input, permuting 100,000 times, the test statistic was 7.06149, and the p value was 0.008. This result shows that the strain level can provide more information than the species level, for metagenomic disease association studies.
